# Phytoplankton Composition and Ecological Status of Lakes with Cyanobacteria Dominance

**DOI:** 10.3390/ijerph19073832

**Published:** 2022-03-23

**Authors:** Małgorzata Poniewozik, Tomasz Lenard

**Affiliations:** 1 Department of Plant Physiology and Biotechnology, Faculty of Natural Sciences and Health, The John Paul II Catholic University of Lublin, Konstantynów 1I, PL-20-708 Lublin, Poland; 2 Department of Animal Physiology and Toxicology, Faculty of Natural Sciences and Health, The John Paul II Catholic University of Lublin, Konstantynów 1I, PL-20-708 Lublin, Poland; tomekl@kul.pl

**Keywords:** ecological status, phytoplankton, PMPL, PSI, Q index, phytoplankton functional groups, phytoplankton morpho-functional groups

## Abstract

Phytoplankton is one of the five biological quality elements used in the assessment of the ecological status of surface waters according to the European Water Framework Directive established in 2000. In this study, we determined the ecological status of three small and shallow lakes in the Polesie Plain, Eastern Poland, by using indices based on phytoplankton assemblages. The predominant phytoplankton of all three lakes were filamentous cyanobacteria, both heterocystous and non-heterocystous, represented by the genera *Aphanizomenon*, *Planktothrix*, *Limnothrix,* and *Planktolyngbya*. We used the Hungarian Q index, German PSI (Phyto-See-Index), and recently developed PMPL (Phytoplankton Metrics for Polish Lakes) for Polish lakes. We compared the results from the calculation of the indices to physicochemical data obtained from the lake water and Carlson’s Trophy State Index (TSI). On the basis of TSI, Gumienek and Glinki lakes were classified as advanced eutrophic, whereas Czarne Lake had a better score and was classified as slightly eutrophic. The trophic state was generally confirmed by the ecological status based on phytoplankton indices and also showed the diverse ecological situation in the lakes studied. Based on the Polish PMPL, Gumienek Lake was classified as having bad status (ecological quality ratio (EQR) = 0.05), whereas Glinki and Czarne lakes were classified within the poor status range (EQR = 0.25 and 0.35, respectively). However, based on the German PSI, the lakes were classified in a different manner: the status of Gumienek and Czarne lakes was better, but unsatisfactory, because they were still below the boundary for the good status category recommended by the European Commission. The best ecological status for the studied lakes was obtained using the Q index: Gumienek Lake with EQR = 0.42 had a moderate status, and Czarne Lake with EQR = 0.62 obtained a good status. However, Glinki Lake, with EQR = 0.40, was classified at the boundary for poor and moderate status. Based on our study, it seems that the best index for ecological status assessment based on phytoplankton that can be used for small lakes is the Polish (PMPL) index.

## 1. Introduction

Phytoplankton is essential for the ecological processes in freshwater ecosystems and reacts very quickly to changes in the physical, chemical, and biological factors [1]. Because eutrophication is a worldwide threat to the well-being of lakes, studies have proposed many methods and attempts to assess the water quality based on phytoplankton species and assemblages. The oldest classification systems, such as the saprobic system, is based on certain organisms that are treated as indicators of water quality in various zones. These classification systems were characterised by excessive randomness where the presence of any species determines the most accurate saprobic class without taking into consideration the quantity of the species or the species assemblages [2,3,4]. The first system based on phytoplankton species was introduced 45 years ago by Tilman and Kilham [5], who used two diatoms (*Asterionella formosa* and *Cyclotella meneghiniana*) as model organisms to obtain information on the natural silicate-phosphate gradient limiting their development. Over the decades, there have been many studies of phytoplankton species as water quality and trophic status indicators e.g., [1,6,7,8,9]. Reynolds et al. [10] presented a new concept, functional groups (FGs) of phytoplankton, which were organised based on morphological and physiological similarities and ecological demands of the species from different systematic levels that demonstrated similar adaptive features [10]. Functional groups are still widely used [11,12,13,14,15,16]. On the basis of FGs, some new ideas have been proposed. Morphological and functional separation within cyanobacteria and algae is a concept leading to the discrimination of 31 morpho-FGs (MFGs) based on several features of these organisms, including motility, the ability to obtain carbon and nutrients by mixotrophy, specific nutrient requirements, size and shape, and the presence of gelatinous envelopes, as well as different life strategies [17]. The FG classification based on morphology was also introduced, wherein phytoplankters were grouped by morphologically similar species regardless of taxonomic affiliation [18]; however, this classification has a rather loose connection with the assessment of lake conditions and ecological status. According to the European Water Framework Directive [19], phytoplankton is one of the main quality components for lake ecological status assessment, and it has been used in a wide range of monitoring surveys, e.g., the Q index for Hungarian [11], PSI system for German [20], PMPL for Polish [21], and PTI for Italian [22] water bodies, as well as the Estonian index for coloured waters [23] and the Brettum index for alpine lakes [24]. Based on phytoplankton and other biological components, assessments are applied primarily to large lakes of more than 50 ha in surface area. There have been no indices dedicated to lakes smaller than 50 ha, although such lakes are very common in nature. Until recently, small water bodies have generally been ignored compared to the attention paid to large water ecosystems [25]. However, such small lakes account for at least a third of the aquatic ecosystem processes on the planet [26]. The aquatic cycles and processes are more intense, complex, and abundant in ponds and small lakes than in larger ones, and they have an undeniable influence on the local climate and biological diversity [26,27,28,29,30].

The aim of this study was to (i) evaluate the ecological status of three small and shallow lakes, (ii) determine the specific taxa for each lake studied, (iii) determine whether the chosen phytoplankton indices applied to small and/or brown-coloured freshwater bodies, and (iv) determine whether the different physicochemical conditions and phytoplankton structure could be explained by functional approaches (FG or MFG).

## 2. Materials and Methods

We chose three lakes located within the Łęczna-Włodawa Plain (Eastern Poland) that are small and shallow (Gumienek, N 51°50′ E 22°93′; Czarne, N 51°48′ E 22°94′; and Glinki, N 51°50′ E 23°55′). The lakes are differentiated in their hydromorphological features. Gumienek Lake is located in a complex of breeding ponds, and it has a small outflow to the Bobrówka River. Czarne Lake is surrounded by the earth dyke, whereas Glinki Lake has inflow and outflow of the Tarasienka River in southeastern part of the lake [31]. General characteristics of lakes of the Łęczna-Włodawa Plain classifies the studied lakes to a group of lakes with maximum depth below 15 m, mean depth below 5 m, but relative depth index above 0.01, and mean slope inclination above 1°30″. These parameters suggest well-developed lake basins with a distinctly inclined bottom [32]. The morphometric and physicochemical characteristics of the lakes are given in Table 1. Studies were conducted from May to August/September 2010 (Gumienek and Czarne lakes) and 2012 (Glinki Lake) in 2-week intervals. Phytoplankton samples were collected at the deepest part of the lakes with the Ruttner water sampler (2.0 L capacity) from the water surface to the depth of 3 m at 1 m intervals and added to one collective sample, preserved using Lugol’s solution. Simultaneously, samples were collected for water chemistry, and colorimetry measurements of dissolved (N-NO_3_, N-NH_4_, P-PO_4_) and total fractions (TN and TP) of biogenic compounds were performed in the laboratory [33]. Secchi disk visibility, light intensity, temperature, pH, and conductivity were measured in situ. Light intensity as a photosynthetic active radiation (PAR) was measured by a Li-Cor 192SA under water quantum flat metre. On this basis, the range of euphotic zone of each lake was calculated. In the laboratory, the samples were analysed with the use of spectrophotometric methods to determine the concentration of chlorophyll-*a* [34]. To assess the trophic status of the studied lakes, we used Carlson’s trophic state index (TSI) and the equations described by Carlson [35]. The variables used for these equations included Secci Disk value (SD) (in metres), chlorophyll-*a* (Chl-*a*) content in the epilimnion (in mg m^−3^), and concentration of total phosphorus (TP) in the epilimnion (in mg m^−3^). The trophic status of the lakes was estimated using the mean TSI values. Phytoplankton numbers were estimated using an inverted microscope and the Ütermohl method [36]. We considered individual alga as the unit (unicell, colony, coenobium, or filament). To calculate phytoplankton biomass, unit counts were converted to biovolumes using the guidelines of Hillebrand et al. [37]. For dominants, we determined the species that accounted for at least 30% of the total biomass/number of phytoplankton. All samples were identified to the species level, if possible. Algal taxa were identified based on published taxonomic keys [38,39]. To assess the ecological status, firstly, we used the PMPL method determined for Polish lakes [21]. Next, we adopted the Q index [11] and PSI system [20] determined for Hungarian and German lowland lakes, respectively, and compared the results obtained using the three indices because of the geographical similarity among the regions. It should be pointed out that the above methods were originally determined for natural lakes larger than 50 ha. In this study, we tested the applicability of these methods to small lakes. To calculate the Q index, we classified all the lakes as type 7, calcareous persistent lakes (Table 1), with an average depth of <4 m and area >0.5 km^2^ [11]. The lakes did not completely match these characteristics, but it was the closest type. The Hungarian method is primarily based on FGs, which means that particular assemblages of algae preferring similar physicochemical conditions of water exist in a particular water body. The groups consist of species with similar morphological and physiological features or ecological demands that may potentially reside in a water body and become dominant. The idea of FGs was proposed by Reynolds [10] and has been modified in other studies and expressed as MFGs according to Salmaso and Padisák [17]. The ecological quality ratio (EQR) for lakes range between 0 and 5 and can be divided into a five-grade classification system: 0–1, bad; 1–2, poor; 2–3, medium; 3–4, good; and 4–5, high.

## 3. Results

### 3.1. Physicochemical Characteristics

The mean values for the measured water properties did not differ widely among the studied lakes (Table 1). The water in all the lakes was alkaline with pH values between 8.1 (Glinki Lake) and 8.4 (Gumienek Lake). There was also no significant difference in conductivity, which ranged from 234 µS cm^−1^ in Czarne Lake to 296 µS cm^−1^ in Gumienek Lake. Water colour was measured only for Glinki Lake, because the water had a strong tea colour and was 234 mg Pt L^−1^, whereas the water of the remaining lakes had a ‘normal’ colour, indicating values of approximately 60–80 mg Pt L^−1^ (unpublished data). The results of the colour test and phytoplankton density affected the water transparency value. Glinki Lake had the lowest water transparency values with a mean value of 0.6 m. The values for the other two lakes were 1.5 m for Gumienek Lake and 2.9 m for Czarne Lake (Table 1). The concentrations of total N (TN) and its soluble fraction, N-NH_4_, were the highest in Glinki Lake, but the concentration of phosphorus (P-PO_4_ and TP) was the lowest. Lower mean values for N (N-NH_4_ and TN) and higher mean values for phosphorus (P-PO_4_ and TP) were noted for the Gumienek and Czarne lakes (Table 1). The trophic status of the lakes was estimated using Carlson’s TSI by considering transparency, chlorophyll-*a*, and TP data. The TSI for Czarne Lake was 52, which was slightly above the boundary for the meso-eutrophic level (50). Thus, this lake was classified as slightly eutrophic. The TSI for Gumienek and Glinki lakes was 62 and 65, respectively, indicating that the lakes were in the eutrophic status range (50–70) and were classified as advanced eutrophic.

### 3.2. Phytoplankton Assemblages and Dominant Species

**Figure 1:**
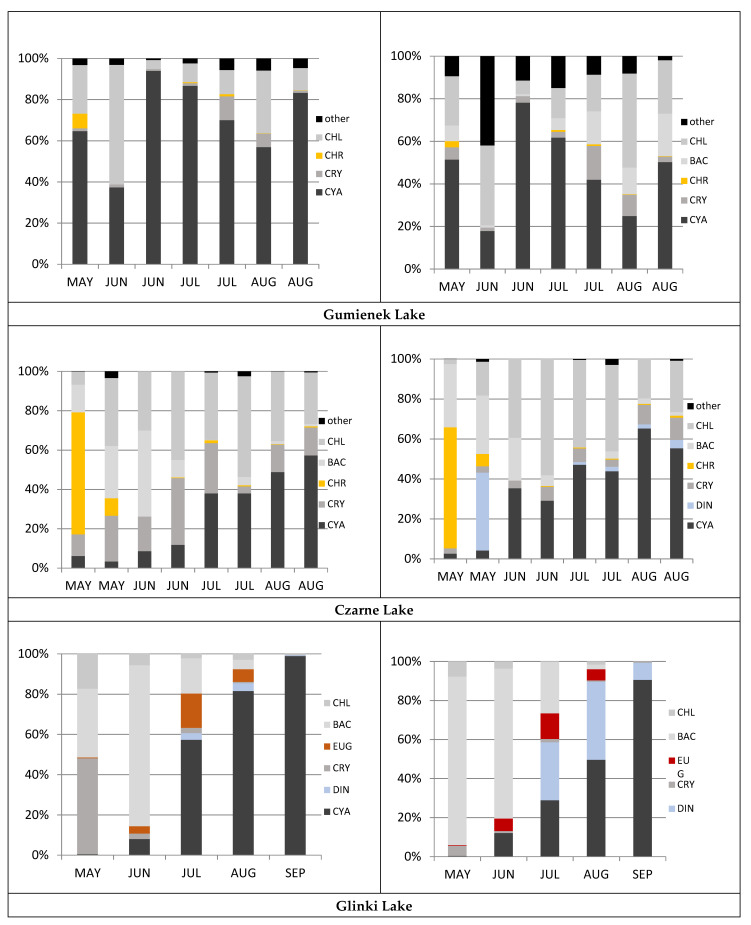
Percentage shares of numbers (left column) and wet biomass (right column) of phytoplankton in the studied lakes in the following month of study. Abbreviations: CHL–Chlorophyta, BAC–Bacillariophyceae, EUG–Euglenophyceae, CHR–Chrysophyceae, CRY–Cryptophyceae, DIN–Dinophyceae, CYA–Cyanophyceae, MAY–May, JUN–June, JUL–July, AUG–August, SEP–September.

In the studied lakes, a total of 178 taxa were identified. These taxa represented the main phytoplankton groups, with the green algae being the most diverse. Considering the numbers and biomass, the main phytoplankton taxonomic group in all three lakes was cyanobacteria. In Czarne and Glinki lakes, the numbers and total biomass of cyanobacteria ranged from several percent at the beginning of the vegetative period to 50–60% and from 90% to nearly 100% of the total, respectively, by the end of the vegetative period (Figure 1). In Czarne Lake, the numbers were 98.5 × 10^3^ indiv. L^−1^ in May and increased to 2895.5 × 10^3^ indiv. L^−1^ by late August 2010. The numbers of cyanobacteria in Glinki Lake were low in May (25 × 10^3^ indiv. L^−1^) but were very high at the beginning of June (433–7387 × 10^3^ indiv. L^−1^). In both lakes, filamentous taxa dominated the cyanobacteria. In Czarne Lake, heterocystous *Aphanizomenon gracile* dominated, although its numbers were not overwhelmingly high (2590 × 10^3^ indiv. L^−1^) by the end of August. Glinki Lake had more species, representing a relatively high percentage of the total number. Initially, *Planktolyngbya limnetica* and *Planktothrix agardhii* represented similar percentages, whereas later the phytoplankton assemblage was predominated by *Cuspidothrix issatschenkoi* in July (827 × 10^3^ indiv. L^−1^) and *Aphanizomenon gracile* from August to the end of September (5121 and 4314 × 10^3^ indiv. L^−1^, respectively). The groups of phytoplankton other than cyanobacteria also reached a significant percentage in Glinki Lake. In the spring, flagellates and diatoms were numerous. In May and June, *Cyclotella meneghiniana* and *Aulacoseira granulata* var. *angustissima* were abundant (1950 × 10^3^ indiv. L^−1^ and 4117 × 10^3^ indiv. L^−1^, respectively), whereas in July and August a high percentage of the biomass was of *Ceratium hirundinella* (30% and 40%) (Figure 1). Chrysophytes including *Dinobryon sociale* (Chrysophyceae) accounted for 45% of the total numbers in the spring in Czarne Lake (Figure 1). They were accompanied by *Cyclotella* sp. and *Fragilaria crotonensis* and several species of chlorophytes; however, none of these reached an important percentage. It was notable that the numbers of the small cryptophyte *Plagioselmis nannoplanctonica* were maintained at approximately 15–20% of the total numbers throughout the study period (Figure 1). Similarly, in Glinki Lake, cryptophytes were represented by *Rhodomonas pusilla*, which was responsible for 45% of the spring dominance in that group of flagellates. Gumienek Lake presented a different pattern, with only cyanobacteria dominating, at 1950.5 × 10^3^ indiv. L^−1^ in June to 42,153 × 10^3^ indiv. L^−1^ in August. The mean value of the numbers in Gumienek Lake, which is more than 24 million indiv. L^−1^, was almost 10 times higher than that in Czarne Lake and four times higher than that in Glinki Lake (Table 1). In Gumienek Lake; among the dominant cyanobacteria, *Limnothrix redekei* was present at high numbers during the entire period, and *Aphanizomenon gracile* and *Planktolyngbya limnetica* were present at high numbers during the second half of the study. Other species included chlorophytes, which accounted for 15–20% of the total phytoplankton during the study (Figure 1). Among the taxa of small green algae and desmids, *Tetraedron minimum*, *Elakatothrix lacustris*, and *Monoraphidium minutum*, and *Cosmarium depressum* were the most abundant, respectively.

Total wet biomass of cyanobacteria and algae was the highest (mean, 20.38 mg L^−1^) in the eutrophic Gumienek Lake and the lowest (4.74 mg L^−1^) in the slightly eutrophic Czarne Lake. The values of wet biomass did not correspond with those of chlorophyll-*a* concentration during particular months or the entire study period (Table 1). Algae and cyanobacteria distribution in terms of numbers was related to the total biomass of phytoplankton. The main difference was the presence of dinophytes and cryptophytes in the phytoplankton assemblage in the studied lakes. High numbers of cryptophytes were not reflected in the biomass values. They were abundant during the spring period in Czarne and Glinki lakes, at up to 906 × 10^3^ and 2743 × 10^3^ indiv. L^−1^, respectively, whereas their biomass values were low, at 0.32 and 0.37 mg L^−1^. Conversely, dinophytes consisting of *Peridinium aciculiferum* in Czarne Lake and *Ceratium hirundinella* in Glinki Lake did not constitute a significant percentage in terms of numbers, but the total biomass was as high as 40% in Gumienek Lake and 30–40% in Glinki Lake (Figure 1).

**Figure 2:**
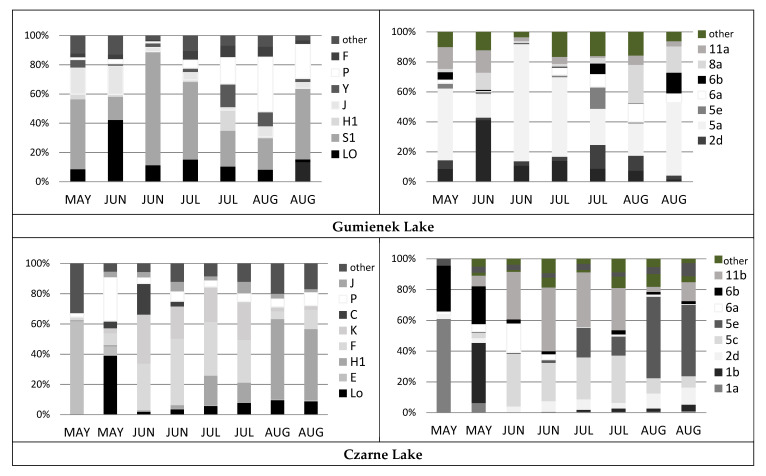

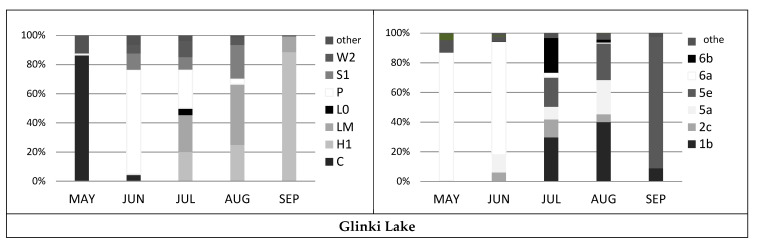
Percentage shares of functional (left column) and morpho-functional (right column) groups—FGs and MFGs in the studied lakes in the following month of study. Abbreviations: MAY–May, JUN–June, JUL–July, AUG–August, SEP–September.

Functional group diversity differed in the studied lakes. In the eutrophic Gumienek Lake, the S1 group was the highest with values between 16% in early June and almost 80% at the end of June. The S1 group was represented by thin solitary filamentous cyanobacteria without heterocysts, including *Limnothrix redekei* and *Planktothrix agardhii¸* which occurred from spring to late summer. The group numbers intensified in summer to include *Planktolyngbya limnetica*. At the beginning of the study season (early June), group Lo was abundant, with 40% (*Peridinium aciculiferum* was the main representative of this group), whereas at the end of the season, group P, with the diatom *Aulacoseira granulata* and green alga *Closteriopsis longissima* represented a significant proportion (40%) (Figure 2). Furthermore, the other groups, J, H1, Y, and F (Figure 2), mainly represented those in typical shallow lake conditions, i.e., enriched and exposed, with relatively low mean depth and satisfying the mixing criterion in the epilimnia of stratified lakes. Similar to Gumienek Lake, in Glinki Lake, the groups P and L_M_ exhibited significant percentages during the study period. However, the order of the presence of these groups in time was the opposite. Group P, represented by *Aulacoseira granulata* var. *angustissima*, accounted for 75% in June and maintained a significant percentage up to late July, whereas group L_M_, with *Ceratium hirundinella*, occurred at a high percentage in July and especially in August, at 25–40% (Figure 2). Apart from *A. granulata* var. *angustissima*, there was another diatom species that should be mentioned. At the beginning of the study season, *Cyclotella meneghiniana*, forming group C, was abundant at 1950 × 10^3^ indiv. L^−1^ and biomass of 6.12 mg L^−1^. At the beginning of June, a switch between diatoms was noted (*C*. *meneghiniana* switched with *A*. *granulata* var. *angustissima*). An important group that began to form in July and had the highest percentage in September was H1. The group comprised *Aphanizomenon* species: in July by *A*. *issatschenkoi* (*Cuspidothrix issatschenkoi*) and in August and September by *A*. *gracile*, with the highest value reaching more than 80% (Figure 2). A different pattern of FGs was noted for Czarne Lake. Several FGs were identified that reached at least 10% in any of the studied months. Apart from what is typical for these lakes, i.e., groups such as H1, Lo, and P, group F was also present during almost the entire study period and was mostly represented by moderately sized gelatinous green algae, such as *Coenococcus planctonicus* and *Quadrigula closterioides*, with the maximum development during June to July. Group K also maintained a percentage during all the study months, and *Aphanocapsa incerta* was the only representative of the group, with an average of approximately 15%. At the beginning of the season, group E, with different species of the genus *Dinobryon*, represented nearly 60% (Figure 2); *Dinobryon sociale* was the most abundant. Groups H1 and P were represented by *Aphanizomenon gracile* and *Fragilaria crotonensis*, respectively. The latter occurred at a significant percentage during May, whereas the former began to occur at the end of June, and its percentage was maintained until the end of August, reaching the highest value of approximately 50% (Figure 2). Taking into consideration the MFGs in Lake Gumienek, group 5a (thin filaments: Oscillatoriales) was the most significant. Filamentous cyanobacteria occurred at the highest percentage in the second half of June but were clearly visible during the entire study period. They were accompanied by groups 1b (large dinophytes: represented by *Ceratium hirundinella*) in June and 8a (large unicells: represented mostly by desmids of *Closterium* and *Cosmarium*) at the end of summer, in August (Figure 2). In Czarne Lake, three MFGs were the most abundant: 5c—other large colonies (mostly non-vacuolated Chroococcales), 11b—Chlorococcales–gelatinous colonies (occurring in the middle of the study period), and 5e—Nostocales (occurring near the end). At the beginning of the study in May, there were different representatives, including 1a (large chrysophytes/haptophytes), 1b (large dinophytes), and 6b (large pennates). Glinki Lake had the clearest dominant MFGs. May and June were completely dominated by group 6a (large centrics) represented by *Aulacoseira* species. The beginning of September was dominated by 5e (nostocales), represented by *Aphanizomenon* species. Species present during the summer (July and August) formed more diversified assemblages, and several groups exhibited a considerable percentage, including 1b, 2c, 5a, 5e, and 6b, with *Ceratium hirundinella*, *Trachelomonas volvocinopsis*, *Planktothrix agardhii*, *Aphanizomenon gracile,* and *Fragilaria ulna*, respectively, as the dominant species (Figure 2).

### 3.3. Phytoplankton-Based Indices and Ecological Status of the Studied Lakes 

**Figure 3:**
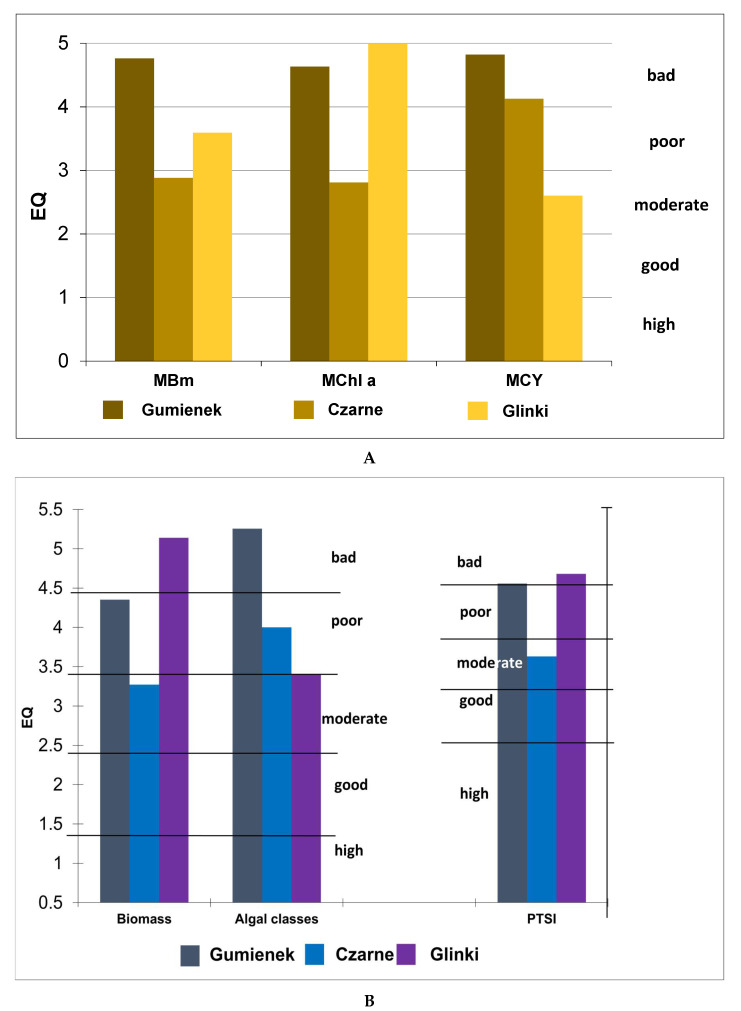

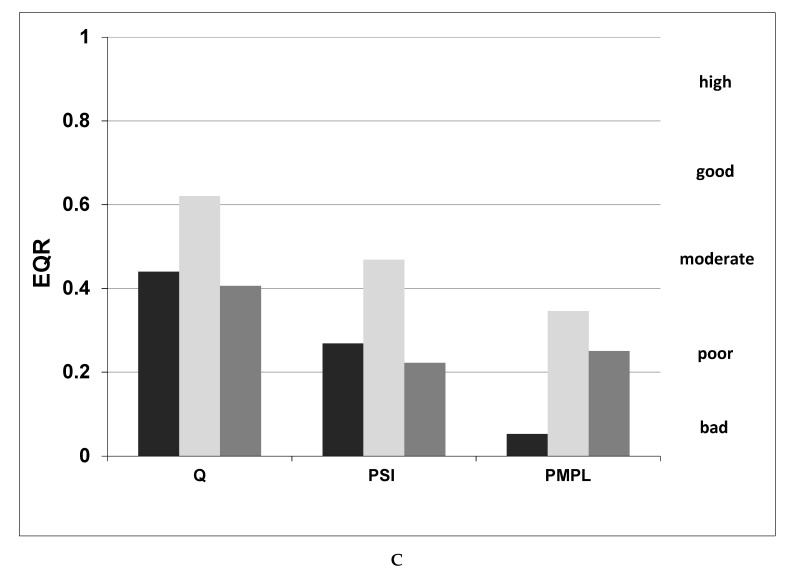
**A**. Ecological Quality (EQ) values and status class assessment according to PMPL metrics of lakes ecological status assessment (Polish method). Abbreviations: MBm–Metric of total biomass of phytoplankton, MChl a–Metric of chl-*a* concentration, MCY–Metric of biomass of cyanobacteria. **B**. Ecological Quality (EQ) values and status class assessment according to German metrics of lakes ecological status assessment. Abbreviation: PTSI–Metric Phytoplankton Seen Index. **C**. Ecological Quality Ratio (EQR) based on Q, PSI, and PMPL indices calculated for the studied lakes. Abbreviations: Q–index for Hungarian lakes, PSI–index for German lakes, PMPL–index for Polish lakes.

The calculated ecological status of the studied lakes based on the chosen indices showed different values. The PMPL index indicated rather poor results. Based on this index, the worst status (bad) was for Lake Gumienek, whereas Czarne and Glinki lakes were characterised by poor status. For the PMPL index, the cyanobacteria biomass in the phytoplankton assemblage was one of the metrics that affected the final results. In the case of Gumienek Lake, all three metrics (total biomass of phytoplankton, MBm; chl-*a* concentration, MChl-*a*; and biomass of cyanobacteria, MCY) indicated a bad status for the lake with values of 4.76, 4.63, and 4.82, respectively (Figure 3A). Czarne Lake had a better status. Based on two metrics, MBm and MChl-*a*, the lake was placed within the moderate status (Figure 3A). However, the third metric, MCY, had a value of 4.13, which lowered the total assessment status of Czarne Lake to poor (EQR = 0.35) (Figure 3C). Apart from *Aphanizomenon gracile*, which occurred at the highest abundance and was responsible for the high numbers and biomass percentage, the cyanobacteria assemblage was quite diverse. Other filamentous species, including *Limnothrix planctonica*, *Planktolyngbya limnetica,* and *Anabaena* sp., and coccal species, including *Aphanocapsa incerta*, *Snowella litoralis*, *Chroococcus limneticus,* and *Chroococcus turgidus*, were identified. Glinki Lake was an interesting example, because it showed different results for the PMPL metrics (Figure 3A). Based on MBm and MCY, Glinki Lake was recognised as being in poor or moderate condition, respectively, but MChl-*a* with the highest value (5, bad status) contributed to the final score of poor status, with EQR = 0.25 (Figure 3C).

Unlike the Polish index (PMPL), the German PSI, which is calculated based on several metrics, classified the lakes differently. The status for both Gumienek and Czarne lakes was better, but still unsatisfactory, because the status remained below the boundary of the good status recommended by the European Commission. For Gumienek Lake, the EQR was 0.27, whereas for Czarne Lake, it was 0.47. For Glinki Lake, the EQR (0.22) was at the boundary of the poor status (Figure 3C). The difference among the lakes was observed for two metrics of PSI, ‘biomass’ and ‘algal classes’. The first was based on the total biomass of phytoplankton and total chlorophyll concentration and resulted in a moderate status for Czarne Lake, poor for Gumienek Lake, and the worst (bad) for Glinki Lake (Figure 3B). The algal classes metric, which is calculated based on the biomass of taxonomic phytoplankton groups, resulted in lower statuses for Gumienek and Czarne lakes (one class lower) but unexpectedly placed Glinki Lake within the boundaries of the moderate status, although algal classes value was close to 3.5, which was the boundary value between moderate and poor (Figure 3B). Different status of the studied lakes was on the basis of the third PSI metric, PTSI, which is calculated based on a comparison of the calculated value to the reference value for a particular lake type. According to this metric, Gumienek and Glinki lakes reached a value of approximately 3.25, which is the boundary value between poor and bad (Figure 3B). Czarne Lake was classified to be of moderate status, similar to that using the metric ‘biomass’ (Figure 3B).

## 4. Discussion

### 4.1. Phytoplankton Composition

In 2000, the European Union established the Water Framework Directive (WFD) [19], wherein the recommendation to assess the ecological status of surface waters in Europe was featured. Ecological status is determined by comparing the current status of a lake to the status in the reference condition, i.e., a condition without disturbances caused by human activity [41]. Phytoplankton is one of the five biological quality elements proposed by the WFD to calculate the ecological status of a lake and also includes macrophytes, phyto- and zoobenthos, and fish assemblages.

Phytoplankton composition and its abundance depend upon many factors, such as catchment and self-supply, natural hydrological and climatic conditions, the morphometric features of the lake, and water movement. According to the TSI, all the lakes were eutrophic, although Czarne Lake was the least fertile. The high trophic status of the lakes has been maintained for many years [42,43], and Gumienek Lake has typically been more eutrophic than Czarne Lake. Glinki Lake has been maintained at the same level [42]. The fertility of the studied lakes was caused by many factors. They are largely surrounded by fields under cultivation and farms. Agricultural land use intensifies the use of mineral fertilisers, which affects nutrient availability for macrophytes, algae, and cyanobacteria. This is especially important during spring when runoff from the catchment is the most intense and near aquatic ecosystems where photosynthesising microorganisms, especially cyanobacteria, can intercept and utilise the nutrients. Furthermore, the studied lakes are affected by the drainage system that was initiated in the wetland areas of the Lublin Plain in the 1950s. Many natural lakes in the region were converted into storage reservoirs, while others were partially transformed for use of embankments on their shoreline [44,45]. Consequently, the number and area of wetlands, including that of lakes, has been modified to a great extent. Drainage of wetlands contributed to increased runoff of surface waters and decreased groundwater levels, and thereby lead to the transformation of peatlands into anaerobic peat bogs [46]. All these changes, in turn, have had a negative effect on the entire hydrological cycle, as has been observed in the Marne reservoir in northern France [47]. Furthermore, drainage of wetland ecosystems accompanied by intensified eutrophication resulting from agricultural nutrient inputs can lead to degradation of valuable habitats and result in decreased biodiversity [48,49]. The eutrophic status of the studied lakes was proven by using phytoplankton composition. Species and assemblages were present that were typical of fertile waters. The phytoplankton of the studied lakes was predominated by cyanobacteria, especially filamentous species, both heterocystous and non-heterocystous. Additionally, in Czarne Lake, coccal species were also significant in both number and biomass. The dominant species in these lakes belonged to the genera *Aphanizomenon* and *Planktothrix*/*Limnothrix*. These species have numerous adaptations and life strategies that help them to compete successfully against other photosynthesising organisms [50]. *Aphanizomenon* species are considered capable of tolerating a wide range of environmental conditions. They are known to develop well at temperatures ranging from 15 °C to 30 °C; *Aphanizomenon gracile*, which was a permanent component in the studied lakes, can grow even when the temperature is approximately or below 10 °C [51]. Additionally, light intensity is an important factor for *Aphanizomenon* species growth, and in general, for the development of filamentous cyanobacteria. This may provide a competitive advantage under situations of reduced light, as is often the case below the surface scum [52]. We observed such a situation, wherein a dense, green cover of algae and cyanophytes was floating during the entire growing period (Glinki Lake) or during the summer months (Gumienek Lake). The light deficiency below the covering still seemed favourable for *Aphanizomenon gracile* and *Cuspidothrix issatschenkoi* development in the studied water bodies, as they reached high numbers (up to 5121.4 × 10^3^ indiv. L^−1^) and biomass (up to 4.5 mg L^−1^) in the summer months. Apart from the physical properties of water, nutrients play a significant role in cyanobacteria development. The lakes we studied can be called N-rich, because the mean values of TN were from 2 to approximately 4 mg L^−1^. These conditions supported the growth of diazotrophic species, such as *Aphanizomenon* and *Cuspidothrix* species [52,53]. Although they are able to fix atmospheric N_2_, they prefer to take it up from the water, if available (or use combined N derived from the atmosphere and dissolved water). This may occur because of the potentially huge energetic costs of N_2_ fixation by using their own heterocysts [54,55]. The intensive development of these taxa may also be supported by P acquisition, because they can utilise inorganic P dissolved in the water, as well as organic P from enzymatic activity involved in the uptake of organic P from either intracellular or extracellular sources [52]. The phenomenon of the occurrence of *Aphanizomenon* species and other filamentous cyanobacteria that could acquire P from different sources was observed in the Baltic Sea after a period of P deficiency [56]. *Planktothrix* species form dense populations in the metalimnetic zone of stratified lakes [57,58], possibly because their requirements for irradiance and temperatures are lower than that of other phototrophic organisms [58,59]. Among cyanobacterial species, *Planktothrix* tends to tolerate a wider range of temperatures than that tolerated by *Microcystis*, *Anabaena,* and *Aphanizomenon* [60,61]. However, in experimental culture studies, the optimum temperature for the growth of *Planktothrix* was between 20 °C and 30 °C [60,62]. Apart from the metalimnion layer, *Planktothrix agardhii* can flourish in the epilimnion during the stratification period [63], and also during the autumn mixing; hence, this species has been shown to occur in both of these periods [64,65]. *Planktothrix agardhii* is a frequent component, often accompanied by the non-toxic *Limnothrix redekei* and *Planktolyngbya limnetica* or toxic *Aphanizomenon* or *Cylindrospermopsis* species in shallow lakes with turbid waters across Europe, e.g., in Poland [66], Germany [67,68], France [69], and Spain [70]. In our study, we observed the composition of species, excluding those of *Cylindrospermopsis*. In combination with polymixis, light and nutrient supply seemed to be key factors for species composition. Because the above species present highly effective adaptations to life conditions, as well as protection against grazing by zooplankters owing to the large size of filaments or the formation of huge aggregations, they are at an ecological advantage relative to other phytoplankton species. The FGs and MFGs of phytoplankton had eutrophic characters in the studied lakes. The most abundant FGs in the study period were S1 and H1. Group S1, composed of *Planktothrix* and *Limnothrix* species, is typical of the highly light-deficient conditions of water bodies with turbid mixed layers [10]. A high percentage of group H1 in the studied lakes represented by dinitrogen-fixing nostocaleans, *Aphanizomenon* species, and *Cuspidothrix issatschenkoi* is characteristic of mixing, poor light, and water with low P [10]. The groups S1 and H1 were also at a high percentage in a floodplain lake under similar conditions [71]. Moreover, we showed that these groups were accompanied by the group Lo at a very high percentage. Our results are consistent with those of Nixdorf et al. [59], who showed that group S1 had the most successful association during the long-term investigation of shallow and turbid lakes in Germany. This regular steady state in eutrophic shallow lakes was explained as a result of adaptations to a resilient and extreme environment. The pool of species that can inhabit or survive in that environment should be considered through a comparison of habitat properties [59]. The permanent dominance of Oscillatoriales during summer and autumn is often reported for eutrophic lakes in central Europe [72]. They were also often found with the group H1 in different parts of the world [73,74,75,76]. The species belonging to FGs S1 and H1 often compete and occur in seasonal succession because of their similar life strategies. The succession depends mostly on inorganic N supply, disturbance patterns, and sudden temperature changes [77].

### 4.2. Phytoplankton-Based Indices and Ecological Status of the Studied Lakes

In our study, the eutrophic/hyper-eutrophic status of the studied lakes was proven based on the methods used in previous long-term studies [42,43]. The raw physicochemical data of these previous studies, as well as biological components such as macrophytes, which were abundant in these lakes [42,43], support the trophic status. Additionally, the Carlson’s TSI calculated during our study pointed towards the eutrophic status of the studied lakes. Such a status was also supported by phytoplankton-based indices. In general, although some differences were noted, the PMPL and PSI did not show different results in this study. The Q index classified the lakes as having better conditions than did the other indices and by the other data, e.g., phytoplankton composition and physical–chemical properties of water. Methods of lake status assessment had been evolving for several years before the rules of WFD were introduced. There are many studies of European lakes e.g., [78,79,80,81,82], but in Poland, this type of research, wherein the ecological status of lakes is assessed, is not common, especially based on phytoplankton data. The PMPL index was specifically developed for Polish lakes and is currently still being tested. There are some studies of large-surface-area lakes [83,84], as well as small lakes of less than 50 ha [15,16,85]. In our study, as well as in previous studies, the results based on the PMPL, PSI, and Q indices were not consistent. The most similar results that we derived were by using the Polish (PMPL) and German (PSI) metrics. The PSI and especially the PMPL indices were strongly dependent on the total phytoplankton biomass and, in contrast to the Q index, on cyanobacteria biomass. This is why lakes with cyanobacteria dominance, as is the case for Gumienek and Glinki lakes, usually reflect a poor ecological status. The Q index is based on the biomass of particular Reynold’s FGs, and each of them was ascribed a factor number (from 0 to 5, depending on the lake type) that reflects the influence of the FG on the ecological status. In this manner, we can hypothetically assume that a water body with 48% cyanobacteria, constituting group S1 with an F factor value of 0, and 62% dominance of euglenophytes, constituting groups W1 (*Euglena*, *Phacus*, and *Lepocinclis*) and W2 (*Trachelomonas* and *Strombomonas*) with F factor value of 5, would probably have physical and chemical parameters indicating advanced eutrophy, because filamentous cyanobacteria, such as *Limnothrix* and *Planktothrix* species, and euglenophytes are indicators of this trophic status [9,10,20,86,87,88]. However, using the Q index, we would obtain a high score (Q = 3.1), which would characterise the water body as having a good ecological status. Thus, we conclude that the results we obtained when assessing the studied lakes according to the Hungarian method were higher by at least one class because of the presence of species with a high F factor value. In Glinki Lake, we obtained a high percentage of *Aulacoseira granulata* (F = 5) during May and June, which is also typical of eutrophic waters [9,10,89]. In Gumienek Lake, we also had group P in July and August, represented by *Aulacoseira granulata* and *Fragilaria crotonensis*, and in Czarne Lake, a high percentage of group F (F = 4) represented by *Coenococcus planctonicus* and group K (F = 5), represented by *Aphanocapsa incerta*, which were representative of shallow, nutrient-rich water columns [86].

In conclusion, for the studied lakes, the ecological status based on the PSI and PMPL indices corresponded to the physicochemical parameters of water and TSI, thereby reflecting the actual conditions of the studied water bodies. It seems that both methods are reliable for assessing the quality of shallow and small lakes. However, the third method, the Q index, was strongly dependent on the determination of the F factor that corresponded to a given lake type based on the lake typology for a given country and seemed to be more sensitive to taxonomic misidentifications of dominant species.

## 5. Conclusions

1. Using three indices basing on phytoplankton data—the Q, PSI and PMPL—we assessed the ecological status of three small and shallow lakes located within the Lublin Plane, Eastern Poland. With one exception, all three indices pointed at a status that was below the boundary for good status recommended by the European Commission. According to the PSI and PMPL indices, the status was worse than using the Hungarian method—the Q index.
2. We claim that the best index that can be used for small lakes is the Polish (PMPL) index. This is due to the fact that small lakes, which undergo eutrophication at a faster pace mainly because of their geomorphological conditions, usually have a high concentration of cyanobacteria, especially in the summer period. The Polish index (PMPL) takes into account the whole cyanobacterial community in lakes as one of the components for calculating the index, thus providing information about the current ecological status of a given lake.
3. The dominant group of all the studied lakes was filamentous cyanobacteria, both heterocytous and non-heterocytous (*Aphanizomenon*, *Planktothrix*, *Limnothrix* and *Planktolyngbya* species), which is a typical group in nutrient-rich lakes.
4. The results we obtained also showed the usefulness of the tested indices for small and shallow freshwaters.
5. The functional approach (functional groups—FGs; or morpho-functional groups—MFGs) seemed to express a diversity of physicochemical conditions in water bodies and the real condition of the studied lakes.

## Figures and Tables

**Table 1:** Morphological, physicochemical, and biological data of the studied lakes.

Morphorogical Data According to Harasimiuk et al. (1998)	Gumienek	Czarne	Glinki
Area (ha)	8.5	24.8	40.9
Maximum depth (m)	7.8	10.3	8.8
Mean depth (m)	3.8	3.7	2.8
Volume (thousands m^3^)	307	915	1342
Catchment area (ha)	21.5	-*	159.7
**Physicochemical data** (mean values and SD–Standard Deviations (±))			
Secchi Disk–SD (m)	1.5 (±0.28)	2.9 (±0.81)	0.6 (±0.16)
Z_eu_ (m)	2.92 (±0.61)	4.38 (±0.79)	1.77 (±0.23)
Water colour (mg Pt L^−1^)	n.d.	n.d.	234 (±49.47)
pH	8.4 (±0.09)	8.2 (±0.10)	8.1 (±0.51)
Conductivity (μS cm^−1^)	296 (±28.60)	234 (±20.10)	246 (±5.85)
Temperature of epilimnion water (°C)	22.9 (±2.95)	21.9 (±2.94)	21.88 (±1.98)
P-PO_4_ (mg L^−1^)	0.015 (±0.002)	0.014 (±0.004)	0.007 (±0.002)
TP (mg L^−1^)	0.111 (±0.005)	0.039 (±0.014)	0.035 (±0.016)
N-NH_4_ (mg L^−1^)	0.26 (±0.042)	0.24 (±0.042)	0.69 (±0.300)
N-NO_3_ (mg L^−1^)	0.72 (±0.490)	0.68 (±0.360)	0.49 (±0.190)
TN (mg L^−1^)	2.53 (±0.590)	2.14 (±0.670)	3.81 (±0.490)
**Biological data** (mean values)			
Total abundance of phytoplankton (N 10^3^ L^−1^)	24436 (±17577)	2637 (±1369)	5779 (±31.44)
Total biomass of phytoplankton (mg L^−1^)	20.38 (±12.38)	4.74 (±1.44)	8.23 (±1.96)
Chlorophyll *a* (μg L^−1^)	20.7 (±6.99)	10.2 (±7.98)	66.1 (±28.10)
**Data for Q index calculation** (Padisák et al., 2006)	**Gumienek**	**Czarne**	**Glinki**
Type of lake	type 7	type 7	type 7
Hydro-geographical features	calcareous	calcareous	calcareous
Persistency of water	persistent	persistent	persistent
**Data for PSI index calculation**			
VQ—ratio of volume of lake to catchment area	1.43	<1.5	0.84
LAWA lake type [40]	13—lowlands, stratified, VQ < 1.5	13—lowlands, stratified, VQ < 1.5	13—lowlands, stratified, VQ < 1.5
**Data for PMPL index calculation**			
Maximum chlorophyll value (μg L^−1^)	30.7	24.5	113.1
Mean value of Cyanoprokaryota biomass (mg L^−1^)	11.85	2.75	2.65

* Czarne Lake has got no its own natural catchment area as this lake is surrounded by the earth dyke and has no inflow of water.
